# von Hippel-Lindau mutants in renal cell carcinoma are regulated by increased expression of RSUME

**DOI:** 10.1038/s41419-019-1507-3

**Published:** 2019-03-19

**Authors:** Lucas Tedesco, Belén Elguero, David Gonilski Pacin, Sergio Senin, Cora Pollak, Patricio A. Garcia Marchiñena, Alberto M. Jurado, Mariana Isola, María J. Labanca, Martin Palazzo, Patricio Yankilevich, Mariana Fuertes, Eduardo Arzt

**Affiliations:** 1 Instituto de Investigación en Biomedicina de Buenos Aires (IBioBA)—CONICET—Partner Institute of the Max Planck Society, Godoy Cruz 2390, C1425FQD Buenos Aires, Argentina; 2 Departamento de Urología, Hospital Italiano de Buenos Aires, VHL Clinical Care Center, Buenos Aires, Argentina; 3 Departamento de Patología, Hospital Italiano de Buenos Aires, Buenos Aires, Argentina; 4 Departamento de Fisiología y Biología Molecular y Celular, Facultad de Ciencias Exactas y Naturales, Universidad de Buenos Aires, Intendente Guiraldes 2160, Ciudad Universitaria, Pabellon II, 2do Piso, C1428EGA Buenos Aires, Argentina

## Abstract

Renal cell carcinoma (RCC) is the major cause of death among patients with von Hippel-Lindau (VHL) disease. Resistance to therapies targeting tumor angiogenesis opens the question about the underlying mechanisms. Previously we have described that RWDD3 or RSUME (RWD domain-containing protein SUMO Enhancer) sumoylates and binds VHL protein and negatively regulates HIF degradation, leading to xenograft RCC tumor growth in mice. In this study, we performed a bioinformatics analysis in a ccRCC dataset showing an association of RSUME levels with VHL mutations and tumor progression, and we demonstrate the molecular mechanism by which RSUME regulates the pathologic angiogenic phenotype of VHL missense mutations. We report that VHL mutants fail to downregulate RSUME protein levels accounting for the increased RSUME expression found in RCC tumors. Furthermore, we prove that targeting RSUME in RCC cell line clones carrying missense VHL mutants results in decreased early tumor angiogenesis. The mechanism we describe is that RSUME sumoylates VHL mutants and beyond its sumoylation capacity, interacts with Type 2 VHL mutants, reduces HIF-2α-VHL mutants binding, and negatively regulates the assembly of the Type 2 VHL, Elongins and Cullins (ECV) complex. Altogether these results show RSUME involvement in VHL mutants deregulation that leads to the angiogenic phenotype of RCC tumors.

## Introduction

von Hippel-Lindau (VHL) disease is an autosomal dominant disorder with a predisposition to highly vascularized tumors development. The most common VHL manifestations are hemangioblastomas of the central nervous system and retina, clear cell renal cell carcinoma (ccRCC), and pheochromocytomas^1^. It is caused by mutations in the tumor suppressor gene *VHL*^1–3^. VHL protein is the substrate recognition component of the E3 ligase complex, composed of Cullin-2, Rbx1, Elongin B, Elongin C, and pVHL, that participates in the oxygen-sensing system that drives Hypoxia inducible factors alpha (HIF) degradation^4^. When *VHL* gene is mutated and its function is modified or lost, HIF-1/2-α subunits are stabilized and HIF target genes become activated^5,6^. HIF activity is linked to tumor angiogenesis, invasion, cancer metabolic reprogramming, and metastasis^5,7^. Although HIF-1α and HIF-2α have been associated with poor prognosis in many human cancers^5^, in some tumors only one HIF-α subunit is correlated with prognosis. Moreover, in some tumor models the expression of HIF-1-α or 2-α subunits can lead to a divergent outcome^8^, probably caused by differential recruitment of coactivators or corepressors to HIF-targeted gene promoters and activation of non-overlapping transcriptional programs^4,5,8^. In RCCs, which are the leading cause of mortality among VHL patients^6^, cumulative evidence support the role of HIF-2 as the driving force for tumor progression^9^. In this regard, there is increasing number of therapies for RCCs that suppress angiogenesis through targeting HIF-VEGF signaling^9,10^. Nonetheless, the resistance to this therapy remains a major issue^9^ and opens the question on the existence of other factors participating in this process^11^.

Germline VHL missense mutations account for 52% of all VHL-associated mutations and are the most frequent kind found in Type 2 form of this disease^1,2^. The proteins translated from some of missense mutated *VHL* gene retain the functional ability to downregulate HIF^12,13^, suggesting additional mechanisms underlying VHL mutation impact on tumorigenesis^14,15^. Under this landscape, research is focusing in new pathways that might play a role in HIF-VHL deregulation, in order to get new knowledge about the molecular mechanism of VHL disease development^16^.

Originally isolated from highly tumorigenic and angiogenic cells, the product of the *RWDD3* gene, RSUME (RWD domain-containing protein SUMO Enhancer) or RWDD3, increases protein sumoylation^17^, a dynamic ubiquitin-like (Ubl) post translational modification^18,19^. It has been demonstrated that ubiquitin and Ubl pathways regulate diverse cellular processes^18–20^ and its alterations have been implicated in the pathogenesis of cancer^20^. RSUME expression has been reported in organs prone to develop VHL syndrome tumors^17,21^. RSUME is upregulated by cellular stress such as hypoxia (HPX)^17^ and acts as a negative regulator of VHL function in normoxia (NMX) promoting HIF-α stabilization^17,21^. Survival analysis available at The Human Protein Atlas with a recent dataset of Cancer Genome Atlas Research Network (TCGA), shows that 20.07% of the 528 RCC tumors samples studied express elevated levels of RSUME, that correlate with a 23% decrease of patients survival rate (https://www.proteinatlas.org/ENSG00000122481-RWDD3/pathology/tissue/renal+cancer/KIRC).

To gain insight into the mechanisms of the loss of function of Type 2 VHL mutants, we investigated the action of RSUME in the deregulation of HIF-2 by Type 2 VHL mutants. We show that RSUME acts on missense VHL mutants promoting HIF-2α stabilization. RSUME, in a sumoylation independent manner, displaces HIF-2-Type 2 VHL mutants interaction and regulates the loss of function of VHL mutants on HIF-2α regulation, leading to enhanced VEGF action and, thus promoting higher vascularized tumors. RSUME levels are higher in tumor patients with VHL mutations and associated with poor prognosis in RCC tumors. Altogether these results support a key role for RSUME on the early angiogenesis needed for tumor establishment by Type 2 VHL mutants.

## Materials and methods

### Materials

Unless otherwise stated, reagents were obtained from Thermo Fisher Scientific (Waltham, MA, USA) or Sigma Aldrich (Saint Luis, MO, USA).

### Cell culture

COS-7 cells were obtained from ATCC. RCC 786-O cells, defective in VHL expression^3^, were obtained from R. Voest (University Medical Center Utrecht) and EA.hy926 cells (human umbilical vein cell line) from G. Owen (Pontifical Catholic University of Chile)^22^. Cells were cultured as described^21,22^ in Dulbecco’s modified Eagle’s medium (DMEM) (pH 7.3) supplemented with 10% fetal bovine serum (FBS), 2.2 g/l NaHCO_3_, 10 mM HEPES, 4mM l-glutamine, 100 U/ml penicillin, and 100 mg/ml streptomycin. All cells were cultured at 37 °C, 5% CO_2_. Cells were regularly tested for Mycoplasma.

For hypoxia^17,21^, cells were incubated in DMEM 2% FBS at 1% O_2_, 5% CO_2_, 94% N_2_ using a hypoxic chamber ProOx Model 110 (BioSpherix, Parish, NY, USA). For MG-132 treatment^21^, cells were incubated in DMEM 2% FBS with 5 μM MG-132 for 6 h.

### Plasmids and transfections

The following plasmids were kindly provided and described as follows pCEFL, O. Coso; Flag-pcDNA3 and Flag-VHL, S. Lee; HA-HIF-2α, W. Kaelin; pBI-GLV4R HRE-LUC, E. Van Meir; V5-Ubc-9 and 6xHis-SUMO-2, R. Hay; siRSUME and siScramble;^17^ shRSUME and shScramble (#KH14185N, SABiosciences, Hilden, Germany);^21^ V5-RSUME, V5-RSUMEY61A/P62A were described previously^17^. Flag-VHL_Y112H_, Flag-VHL_R167Q_, Flag-VHL_L188V_, and Flag-VHL_K171R_ mutants were generated with directed mutagenesis PCR standard protocol and cloned in frame into Flag-VHL plasmids with *Not*I and *Hin*dIII restriction enzymes. After confirming VHL mutations by DNA sequencing, Flag-VHL_Y112H_, Flag-VHL_R167Q_, Flag-VHL_L188V_, primers containing missense mutation were used in a PCR to generate Flag-VHL_Y112H/K171R_, Flag-VHL_R167Q/K171R_, and Flag-VHLL_188V/K171R_ plasmids.

Flag-VHL_K171R_/shScramble, Flag-VHL_K171R_/shRSUME, Flag-VHL_L188V/K171R_/shScramble, and VHL_L188V/K171R_/shRSUME were obtained as described previously^21^. Correct sequence was confirmed for all generated plasmids.

Transfections with lipofectamine reagent were performed following the manufacturer indications, as previously described^17^.

Stable clones were obtained as described^21^, RCC 786-O VHL-negative cells were transfected with Flag-VHL_Y112H/K171R_-shScramble/shRSUME, Flag-VHL_R167Q/K171R_-shScramble/shRSUME, Flag-VHL_L188V/K171R_-shScramble/shRSUME or pcDNA3-Flag-VHL_K171R_-shScramble/shRSUME. Cells were selected with Geneticin (G418) at a concentration of 600 ng/ml. After selection, cells were cultured in DMEM medium containing 300 ng/ml Geneticin. Mutated VHL and RSUME expression levels were checked by western blot (WB) and RT-PCR, respectively.

### Western blot assay

WB analysis was performed as described previously^17^. Cells lysates were prepared in 2x Laemmly buffer and separated in sodium dodecyl sulfate polyacrylamide gel electrophoresis (SDS-PAGE). Membranes were incubated with specific primary antibodies, followed by incubation with HRP-conjugated secondary antibodies (Bio-Rad Laboratories, Hercules, CA, USA). Developing was performed with the SuperSignal West Dura kit according to manufacturer’s instruction (Pierce Biotechnology, Waltham, MA, USA) using G:BOX-CHEMI-XT4 (Synoptics Ltd., Cambridge, United Kingdom). The following antibodies were used as already described:^17^ anti-V5 (1:3000; Abcam, Cambridge, United Kingdom); anti-β-actin (C4) (1:2000; Santa Cruz Biotechnologies, Dallas, TX, USA), anti-Flag (M2) (1:5000); anti-HA (C11) (1:2000; Covance, Princeton, NJ, USA), anti-HIF-2α (1:1000; Novus Biologicals, Littleton, CO, USA); anti-RWDD3 (1:1000; Abcam).

### Luciferase assay

Cells were transfected with HRE-LUC and RSV-β-gal, coding for the bacterial β-galactosidase gene under the control of the viral RSV promoter, to standardize the results. After 24 h, cells were washed with Phosphate Buffered Saline (PBS) and lysed with Passive Lysis Buffer (Promega, Madison, WI, USA). Luciferase activity was measured using the Luciferase detection kit (Promega) with a Junior Luminometer (Berlthod, Bad Wildbad, Germany), as previously described^17^.

For β-galactosidase activity, 100 μl of β-galactosidase buffer (100 mM Na_2_HPO_4_/NaH_2_PO_4_ pH 7.4, 1 mM MgCl_2_, 50 mM β-mercaptoethanol and 0.66 mg/ml 2-Nitrophenyl β-d-galactopyranoside (ONPG)) were added to 20 μl of lysates. After incubation at 37 °C, β-galactosidase activity was determined using iMarkTM Microplate Reader (Bio-Rad) at 415 nm.

### Co-immunoprecipitation

COS-7 cells were co-transfected with indicated expression vectors. When indicated, cells were treated with 5 µM MG-132 for 6 h, washed twice with ice cold PBS, lysed on ice with modified RIPA buffer (50 mM Tris-Hcl pH 7.4, 1 mM EGTA, 1% NP-40, 150 mM NaCl), supplemented with 2 mM Sodium Orthovanadate, 1 mM PMSF, and protease inhibitors cocktail (Roche, Basilea, Switzerland). Extracts were immunoprecipitated with the indicated antibodies as previously described^17^. Cell lysates were centrifuged at 12,000 × *g* for 30 min at 4 °C. Supernatants were pre-cleared in RIPA buffer with 15 μl of protein A-agarose beads, followed by immunoprecipitation with indicated antibodies. Twenty-five microliters of protein A-agarose beads were added to cells extracts and incubated for 1.5 h at 4 °C. Beads were collected by centrifugation, washed four times with 500 μl modified RIPA buffer and boiled at 95 °C for 5 min in 2x Laemmly buffer.

### Tandem immunoprecipitation

Cells were washed with PBS, lysed on ice with modified RIPA buffer, and tandem immunoprecipitation was performed as previously described^21^. Cells were washed with PBS, lysed on ice with modified RIPA buffer, and immunoprecipitated with anti-FLAG antibody as described above. Immunoprecipitates were eluted with 3xFLAG peptide 100 μg/ml, in 100 µl of RIPA Buffer, for 1 h at 4 °C. After centrifugation, eluates were subjected to second immunoprecipitation protocol (Tandem immunoprecipitation) using anti-V5 antibody (Abcam). WB analyses were performed with the indicated antibodies.

### 6xHis-SUMO-2 conjugates purification

6xHis-SUMO-2 conjugates purification was performed as described^21^. COS-7 cells were transfected with indicated plasmids. After 48 h, cells were lysed in 6 M guanidine-HCl, 100 mM Na_2_HPO_4_/NaH_2_PO_4_, 10 mM Tris/HCl, 10 mM iodoacetamide, 5 mM imidazole, pH 8. 50 µl of Ni-NTA agarose beads were added and after incubation for 4 h at room temperature, beads were sequentially washed with buffer A (8 M urea, 100 mM Na_2_HPO_4_/NaH_2_PO_4_, 10 mM Tris/HCl, 10 mM iodoacetamide, 5 mM imidazole, pH 8), buffer B (8 M urea, 100 mM Na_2_HPO_4_/NaH_2_PO_4_, 10 mM Tris-HCl, 0.2% triton X-100, 10 mM iodoacetamide, 5 mM imidazole, pH 6.3), and buffer C (8 M urea, 100 mM Na_2_HPO_4_/NaH_2_PO_4_, 10 mM Tris-HCl, 0.1% triton X-100, 10 mM iodoacetamide, 5 mM imidazole, pH 6.3). Elution was performed with 150 mM Tris/HCl pH 6.7, 5% SDS, 200 mM imidazole, 30% glycerol, and 720 nM β-mercaptoethanol, for 20 min at room temperature. After centrifugation, supernatants were directly analyzed by SDS-PAGE and WB.

### Quantitative reverse transcription PCR (qRT-PCR)

Quantitative real-time RT-PCR was performed with cDNA samples from RCC 786-O stable clones. The amplification reactions of 40 cycles were carried out with specific primers for VEGF (upper: 5´ AGCTACTGCCATCCAATCGA 3´ lower: 5´ GGTGAGGTTTGATCCGCATA 3´), and RPL19 (upper: 5′ CAATGCCAACTCCCGTCAGCAGATC 3′; lower: 5′ GTGTTTTTCCGGCATCGAGCCC 3´). SYBR Green qPCR amplifications were performed by Real-time PCR, and the data were analyzed with Bio-Rad CFX Manager software. For each sample, the values were normalized to RPL19 levels.

### Conditioned media from clones

RCC 786-O-derived clones were seeded at 1 × 10^5^ cells/well and maintained under normal culture conditions. After 24 h cells were maintained in DMEM 2% charcoal FBS for 72 h. Conditioned medium was collected, centrifuged at 500 × g for 5 min and supernatants were stored at −80 °C until use.

### Tube formation assay

The angiogenic response to conditioned medium was assessed using an in vitro capillary/tube-like structure formation assay^22^. Ninety-six-well cell culture plates were coated with growth factor-depleted Matrigel (Corning inc., Corning, NY, USA) for 30 min at 37 °C. EA.hy.926 cells were resuspended in RCC 786-O-derived clones conditioned medium and seeded (1.5 × 10^4^ cells/well) onto 96-well cell culture plates coated with Matrigel. For negative control EA.hy.926 cells were resuspended in DMEM 2% fetal bovine charcoal serum. After 18 h of incubation at 37 °C and 5% CO_2_, the capillary/tube-like structures were visualized by light microscopy and were analyzed using NIH ImageJ software. Two independent experiments were performed. For each conditioned media three individual wells were photographed and the tube network was quantitated and expressed as average of tubules from the 6 wells.

### In vivo angiogenesis assay

In all, 10^6^ cells of each RCC 786-O clone were harvested in 90 µl of DMEM and 10 µl of Tripan Blue were added. Cells were intradermally injected (27 G needle) in the right flank of 6–8-weeks male NOD/SCID mice (10^6^ cell/mouse) and vehicle in the left flank. Animals were housed with access to food and water ad libitum in ventilated mouse cages (1–5 mice per cage) at the Institute Animal Services Facility. All animal experimental protocols were approved by the Ethical Committee on Animal Care and Use (CICUAL), University of Buenos Aires, Argentina and performed in compliance with ARRIVE Animal Research guidelines recommendations. Mice for each group (treatment) were selected at random. After 7 days, animals were sacrificed and the skin was removed. Photographs were taken under magnification glass using ZEN software (Carl ZEISS, Oberkochen, Germany). Small and medium vessels were measured using ImageJ software and vessel density was calculated as (number of vessels_cells side_ − number of vessels_vehicle side_)/Area. Trial experiments and experiments done previously were used to determine sample size with adequate statistical power.

For both angiogenesis and tube formation assays data analysis of Fig. 5b–i were performed by two independent blinded observers.

### RCC tumor samples

The clear renal cell carcinoma (RCC) paraffin samples were provided by Hospital Italiano de Buenos Aires (VHL Clinical Care Center of VHL Alliance, USA). This study complies with the June 1964 Declaration of Helsinki and has been approved by the Hospital Italiano ethics committee.

Informed consent was obtained for all patients as part of the Cancer Genome Atlas consortia. All data used in this study were downloaded from public websites after the data were consented for public use.

### Immunohistochemistry

Paraffin embedded samples were cut in slides (5 μm). Deparaffinization and citric acid based antigen retrieval was performed following standard protocols. Tumor tissues were fixed in 4% paraformaldehyde for 5 min and blocked in 5% goat serum with 0.1% (v/v) triton X-100 for 1 h at room temperature. Slides were incubated with RSUME antibody (1:200; Abcam) overnight at 4 °C. After washing, biotinylated anti-rabbit secondary antibody (1:300; Vector Laboratories Ltd., Peterborough, United Kingdom) was added for 30 min at room temperature. The slides were incubated for 30 min with the avidin–biotin–peroxidase complex (Vector Laboratories Ltd.). Color development was performed using 1 mg/ml diaminobenzidine with 0.01% hydrogen peroxide for 8 s. Sections were counterstained with Hematoxiline. Images were acquired at a magnification x20 in a LSM 710 AxioObserver (Carl ZEISS) microscope.

### Bioinformatics analysis

#### Data

The bioespecimen data of somatic variants identified from exome sequencing studies of ccRCC tumors correspond to the TCGA Kidney Renal Clear Cell Carcinoma (Project ID: TCGA-KIRC) of the TCGA Resource Network^23^. The datasets analyzed during the current study are available in the NIH, National Cancer Institute, GDC Data Portal repository, [https://portal.gdc.cancer.gov/projects/TCGA-KIRC]. The VHL mutational analysis from tumors with sequencing data was available and downloaded from Table S1 from Ricketts et al.^24^.

RSUME expression and tumor stage of the ccRCC tumors was obtained and downloaded from The human protein Atlas (https://www.proteinatlas.org/ENSG00000122481-RWDD3/pathology/tissue/renal + cancer/KIRC). Expression data is reported as median FPKM (number Fragments Per Kilobase of exon per Million reads). In order to maintain data consistency, from the complete TCGA Resource Network dataset we only used those 463 patients samples and genes that were present in both The Human Protein Atlas and the Table S1 from Ricketts et al. from the TCGA-KIRC.

#### Survival

The survival Kaplan–Meier analysis for 5-year survival was conducted by The Human Protein Atlas (https://www.proteinatlas.org/ENSG00000122481-RWDD3/pathology/tissue/renal + cancer) from 528 ccRCC patients (KIRC dataset).

#### RSUME expression in ccRCC tumor samples

RCC samples were stratified in two groups according to the presence of VHL mutation (See Additional file 8: Table S1). VHL mutated samples were then stratified in sub-groups according to tumor stage. Total samples for tumor stage IV were further divided according to VHL type of mutation. Sample groups were filtered by removing those samples considered as RSUME expression outliers. The interquartile range (IQR) was used to find outliers defined as expression values that fall below first quartile-1.5 IQR or above third quartile + 1.5 IQR.

### Statistics

As indicated for each experiment ANOVA in a combination with the Scheffé's test, Kruskal–Wallis followed by a Dunn’s test, Student’s *t*-test, or Mann–Whitney *U*-test were performed as statistics assays. Data are shown as mean ± SEM. *P*-values are indicated within each figure.

Data analysis from TGCA dataset was performed using Python 3.6.1. Significance differences of distributions between multiple groups were detected through the one-way ANOVA method. ANOVA assumptions of normality and homoscedasticity of each sample group were evaluated. When any of the assumptions were not satisfied, log transformation has been applied to the dependent variable (RSUME expression in FPKM). ANOVA followed by a post hoc Tukey test was performed in order to assay RSUME expression values comparisons between means of tumor stage groups. *P*‐value of pairwise comparisons between two sample groups’ distributions of RSUME expression values were analyzed by non-parametric Mann–Whitney *U*-test.

## Results

### Expression of RSUME is associated with VHL mutations in RCC tumors

**Fig. 1:**
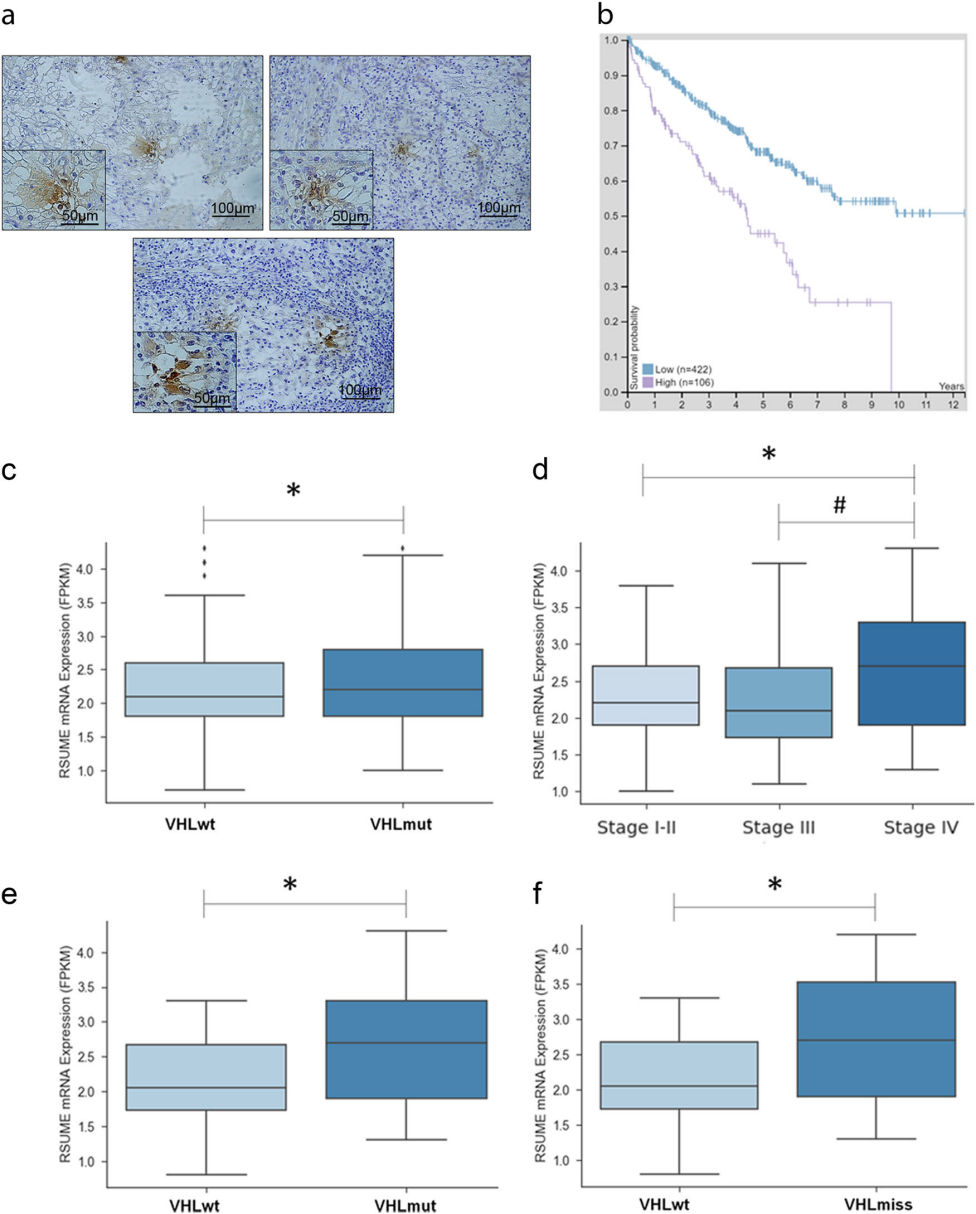
Increased RSUME expression is correlated with VHL missense mutations and poor survival in RCC patients. **a** Representative immunohistochemistry (IHC) staining for RSUME in three ccRCC patient samples. Scale bars 100 µm and 50 µm as indicated. **b** Kaplan–Meier analysis in patients stratified as having high (violet line) RSUME expression or low (blue line) RSUME expression (expression cutoff 2.9 FPKM), publically available from The Human Protein Atlas. **c** Box plots comparison between RSUME mRNA expression in a patient stratification of the Additional file 8: Table S1 data (where VHLwt = 207 samples without mutations in *VHL* gene, VHLmut = 245 samples with mutations in *VHL* gene). *Significant differences between sample groups distribution with a *P* = 0.048, < 0.05 (Mann–Whitney *U*-test). **d** Box plots comparison between RSUME mRNA expression in a patient stratification of the Additional file 8: Table S1 data with mutations in VHL cohort (VHLmut) into tumor stages (where stage I–II represent samples corresponding to tumor stages I and II = 150 samples; stage III represents samples corresponding to tumor stage III = 58 samples; stage IV represents samples corresponding to tumor stage IV = 37 samples). Significant differences between sample group means with a *P* = 0.036 (_*_) and 0.021 (#), < 0.05 (ANOVA and post hoc Tukey tests). **e**, **f** Box plots comparison between RSUME mRNA expression in a patient stratification of the Additional file 8: Table S1 data in stage IV cohort into mutation types (where VHLwt = 34 samples without mutations in *VHL* gene, VHLmut = 37 samples with mutations in *VHL* gene, VHLmiss = 18 samples with mutations missense in *VHL* gene only). *Significant differences between sample groups distribution with a *P* = 0.012 (5e), and 0.027 (5f), < 0.05 (Mann–Whitney *U-*test)

We first studied RSUME expression in tumor samples by immunohistochemical (IHC) staining and performed a bioinformatics analysis of RSUME expression in a ccRCC-TCGA dataset. Figure 1a shows representative staining of RSUME expression in tumor samples of ccRCC patients, that is not uniformly expressed. Kaplan–Meier survival analysis shows that patients exhibiting high RSUME expression within this cohort had significantly worse overall survival compared to those with low RSUME gene expression (Fig. 1b). We next interrogated the TCGA dataset to assess RSUME gene expression levels comparing between patients with VHL mutations (VHLmut) and those without VHL mutations (VHLwt) and observed significant augmented RSUME levels in those with VHLmut (*P* *=* 0.048; Fig. 1c). To assess the impact of RSUME expression in RCC progression, we evaluated the association between RSUME expression levels and tumor stage distribution in VHLmut samples. RSUME expression levels were significantly different, showing a higher level in stage IV than the stages I–II and III (*P* = 0.036 and 0.021; Fig. 1d). Given this increase we next analyzed in more detail the levels of RSUME in stage IV. Patients with VHL mutations show higher RSUME levels compared to those without VHL mutations (*P* *=* 0.012; Fig. 1e). When we interrogated the datasets for the levels of RSUME in patients within this group carrying missense VHL mutations, they also displayed a significant difference with respect to patients without VHL mutations (*P* *=* 0.027; Fig. 1f). Altogether these results indicate an association of RSUME levels with VHL mutations and tumor progression.

### RSUME potentiates VHL Type 2 loss of function

**Fig. 2:**
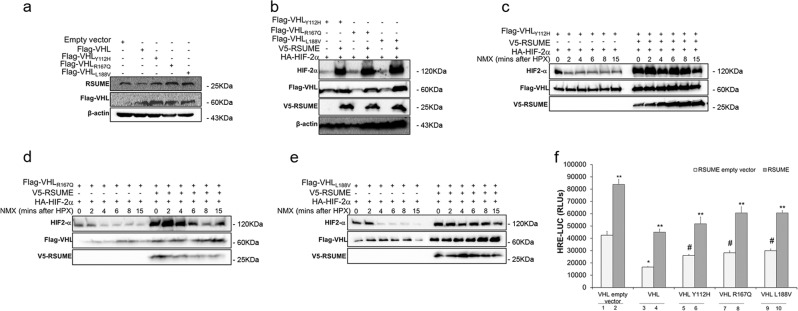
RSUME potentiates VHL type 2 mutants loss of function. **a** RCC-786-O and **b–f** COS-7 cells were transfected with the following vectors as indicated in each panel: 0.5 µg of Flag-VHL variants, 0.5 µg of V5-RSUME, 0.1 µg of HA-HIF-2α, 0.7 µg HRE-LUC reporter vector, 0.5 µg RSV-β-galactosidase or the corresponding empty vectors. **a**, **b** Forty-eight hours post transfection cells were lysed or **c–e** 48 h post transfection cells were incubated for 4 h in hypoxia (HPX) and then harvested in normoxia (NMX) at the indicated times. Cells extracts were analyzed by WB using the indicated antibodies. β-actin was used as a loading control. One representative experiment from three experiments with similar results is shown. **f** Twenty-four hours post transfection Firefly luciferase (LUC) was measured. Each value was normalized by RSV-β-galactosidase activity. Results are expressed as mean ± SEM of triplicates of one representative experiment of three experiments with similar results. *P < 0.05 compared with cells transfected with VHL empty vector (bar 1). **P < 0.01 compared each VHL variant with the corresponding cells transfected with RSUME empty vector. #*P* < 0.05 compared with cells transfected with VHL (bar 3) (one-way ANOVA followed by Scheffé’s test)

To understand the basis for the augmented RSUME levels in patients with VHL mutations (Fig. 1c) we investigated the regulation of RSUME by VHL mutants in a RCC cell line. VHL type 2 representative mutants (Tyr112His, Arg167Gln, and Leu188Val) contrasting to VHL_WT_, do not reduce RSUME expression (Fig. 2a), generating a permissive cellular context for elevated RSUME levels.

RSUME overexpression in the presence of VHL Type 2 mutants causes increased stabilization of HIF-2α (Fig. 2b). In cells previously cultured under HPX and next exposed to NMX, RSUME blocks HIF-2α degradation produced by the incomplete action of VHL mutants (Fig. 2c–e) or by VHL (Supplementary Fig. S1). To verify that HIF-α stabilization due to RSUME action on VHL type 2 mutants produces an increase in HIF transcriptional activity, COS-7 cells were transfected with HIF transcriptional activity reporter (HRE-LUC) in combination with the different variants of VHL. VHL mutants showed decreased but not completely abolished function, as already reported^12,13^, and RSUME significantly increased HIF activity (Fig. 2f), promoting VHL mutants loss of function.

### VHL mutants are sumoylated but RSUME acts independently of their sumoylation status

**Fig. 3:**
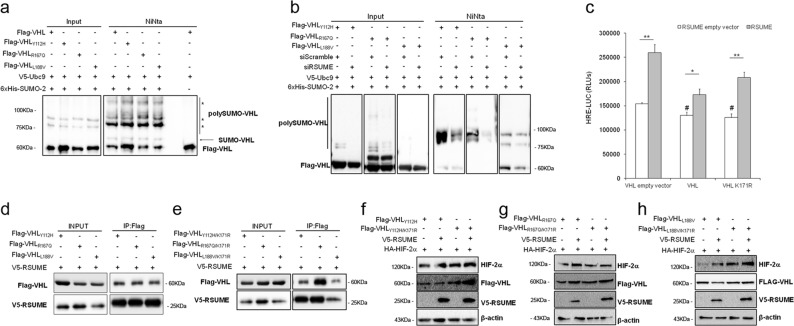
RSUME acts on VHL mutants in a sumoylation independent manner. **a**, **b** COS-7 cells were transfected with the following vectors: 0.6 μg of each Flag-pVHL variant, 0.6 μg of 6xHis-SUMO-2, 0.1 μg of V5-Ubc-9 expression vectors, 20 μM siRNA against RSUME or Scramble as a control. Forty-eight hours post transfection cells were harvested, lysed and an aliquot of the lysates was analyzed by WB (Input). The remaining extracts were subjected to Ni^2+^ affinity chromatography to purify 6xHis-SUMO-2 (Ni-NTA). Purified fraction and Inputs were analyzed by WB using Flag antibody. One representative experiment from two independent experiments with similar results is shown. **c–e** COS-7 cells were transfected with the following vectors: 0.7 μg of HRE-LUC reporter vector, 0.2 μg of RSV-β-galactosidase control vector and/or 0.5 μg of V5-RSUME and/or 0.5 μg of Flag-pVHL or Flag-VHL_K171R_, 0.5 μg of indicated Flag-VHL variants and/or respective empty vector as control. **c** Twenty-four hours post transfection *Firefly* luciferase (LUC) was measured. Each value was normalized by RSV-β-galactosidase activity. Results are expressed as mean ± SEM of triplicates of one representative experiment of three experiments with similar results. *P < 0.05 and **P < 0.01 compared with cells transfected with the corresponding RSUME empty vector, ^#^*P* < 0.05 compared to VHL empty vector without RSUME (one-way ANOVA followed by Scheffé’s test). **d**, **e** Forty-eight hours post transfection cells extracts were Immunoprecipitated with anti-FLAG antibodies. Immunoprecipitated fractions and extract aliquots (Input) were analyzed by WB using the indicated antibodies. One representative experiment from three independent experiments with similar results is shown. **f–h** RCC 786-O cells were transfected with 1.0 μg of Flag-VHL indicated variant and/or 1.5 μg of V5-RSUME expression vectors. Forty-eight hours post transfection cells were lysed and extracts were analyzed by WB using the indicated antibodies. β-actin was used as a loading control. One representative experiment from two independent experiments with similar results is shown

To get knowledge about the mechanism involved, we first evaluated Type 2 VHL mutants sumoylation capacity, as well as RSUME contribution to it. By affinity purification of sumoylated proteins from transiently transfected cells expressing His-SUMO-2, Ubc-9 and the different Type 2 VHL mutants, we observed that VHL mutants are sumoylation substrates (Fig. 3a). Knock-down of endogenous RSUME expression with a small-interfering RNA (siRNA), produced a significant decrease of sumoylation of transiently expressed variants of VHL (Fig. 3b). Thus, RSUME acts on VHL disease representative mutants as a positive modulator of their sumoylation.

We next studied if RSUME needs to promote VHL mutants sumoylation to exert its action. RSUME increased HIF-2α stability both when VHL _WT_ or VHL_K171R_ (VHL defective in sumoylation)^25^ were expressed (Supplementary Fig. S2A, B, C), as well as in cells in which overall sumoylation was inhibited by the expression of the viral protein Gam-1 (Supplementary Fig. S2D), indicating that its action as sumoylation enhancer on VHL is not involved in this mechanism. RSUME sumo-independent inhibition on VHL is accompanied by an increase of HIF transcriptional activity (Fig. 3c).

Given that the consensus site for sumoylation and the Type 2 mutations are near and in order to confirm that RSUME retains its action on VHL mutants when their sumoylation is affected, we generated plasmids expressing both VHL disease variants and the K171R mutation (VHL _Y112H/K171R_, VHL_R167Q/K171R_ and VHL_L188V/K171R_), which showed impaired sumoylation (Supplementary Fig. S3). VHL mutants interact with RSUME (Fig. 3d) and this interaction is not affected by the VHL sumoylation mutation (VHL_K171R_) as double VHL mutants also interact with RSUME (Fig. 3e). In line with this, in RCC 786-O cells transfected with each single mutant or its corresponding sumoylation defective variant VHL_Y112H_ and VHL_Y112H/K171R_ (Fig. 3f), VHL_R167Q_ and VHL_R167Q/K171R_ (Fig. 3g) and VHL_L188V_ and VHL_L188V/K171R_ (Fig. 3h), RSUME overexpression stabilized HIF-2α, potentiating simple and double mutants loss of function (Fig. 3f–h). RSUME action was also confirmed in transfected COS-7 cells (Supplementary Fig. S4A, B, C). In line with a sumoylation independent mechanism both VHL and VHL_K171R_ co-immunoprecipitated with the RSUME_YAPA_ variant, carrying a mutation on a conserved residue of its RWD-domain essential for sumoylation enhancement (Supplementary Fig. S5, lanes 6 and 8).

Altogether, these results indicate that VHL mutants are sumoylated but its sumoylation is not involved in RSUME potentiation of VHL mutants loss of function.

### RSUME acts on VHL Type 2 mutants by decreasing mutant VHL–HIF binding and by disrupting their ECV complex assembly

**Fig. 4:**
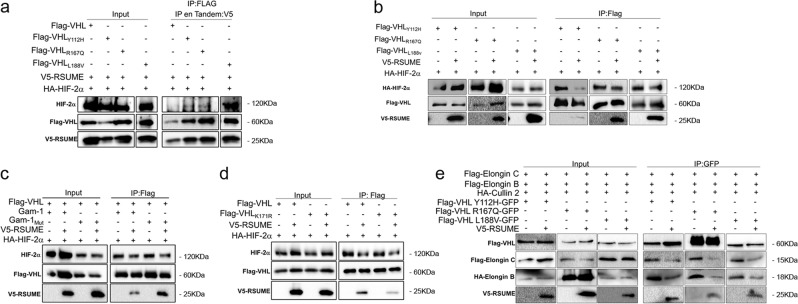
RSUME potentiates VHL mutants loss of function by decreasing VHL–HIF interaction and ECV complex assembly. **a–d** COS-7 cells were transfected with 0.5 µg of Flag-VHL variants, 0.5 µg of V5-RSUME, 0.5 µg of HA-HIF-2α, 0.1 μg of Gam-1, Gam-1_MUT_ control vector. **a** Forty-eight hours post transfection cells were incubated in 2% serum, 5 μM MG-132 for 6 h and lysed. A first immunoprecipitation (IP) using anti-FLAG antibody was performed. Immunoprecipitated fractions were eluted with soluble FLAG peptide and a second IP using anti-V5 antibodies was performed. Immunoprecipitated fractions from the second IP “Tandem IP” and extract aliquots (Input) were analyzed by WB using the indicated antibodies. **b–d** Cells extracts were immunoprecipitated with anti-FLAG antibodies. Immunoprecipitated fractions and extract aliquots (Input) were analyzed by WB using the indicated antibodies. One representative experiment from three independent experiments with similar results is shown. **e** Cells were transfected with 0.5 μg of the indicated Flag-VHL-GFP variants, 0.5 μg of Flag-Elongin C, 0.5 μg of HA-Elongin B, 0.5 μg of Cullin-2 and/or 0.5 μg of V5-RSUME expression vectors. Forty-eight hours post transfection cells were lysed and lysates immunoprecipitated with GFP antibody. Immunoprecipitated fractions and extract aliquots (Input) were analyzed by WB using the indicated antibodies. One representative experiment from two independent experiments with similar results is shown

To explore the molecular mechanism by which RSUME impacts on VHL mutants function, we next addressed whether RSUME would coexist with VHL variants and HIF-2α in a complex. We performed tandem immunoprecipitation (first purification: VHL-associated proteins; second purification: RSUME-associated proteins), showing that in addition to binding to VHL Type 2 mutants RSUME participates in a VHL–HIF-RSUME complex for all Type 2 mutants (Fig. 4a). RSUME decreases the binding of HIF-2α to all disease VHL mutants analyzed (Fig. 4b) even when a slight increase of VHL was observed, providing a molecular basis by which RSUME potentiates their loss of function. Moreover, when sumoylation was inhibited by Gam-1 or in presence of VHL defective for sumoylation, RSUME also impaired VHL–HIF-2α interaction (Fig. 4c, d), further confirming that RSUME-mediated decrease in HIF-VHL mutant binding is the mechanism responsible for HIF stabilization. Furthermore, the interaction of RSUME with VHL type 2 mutants impacts on the ECV complex assembly, affecting VHL variants binding to Elongin C and Elongin B (Fig. 4e).

### RSUME participates in early angiogenesis produced by VHL mutants

**Fig. 5:**
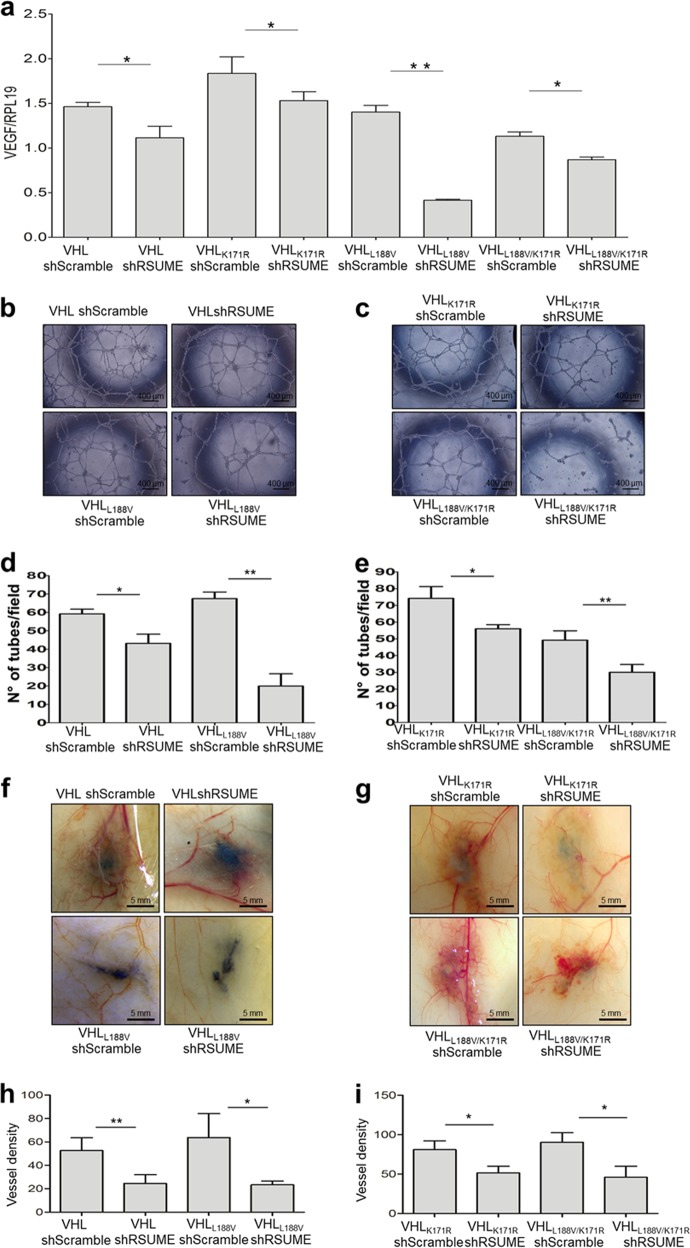
RSUME impacts on VHL mutants function and increases early angiogenesis. **a** RCC 786-O clones were lysed and VEGF mRNA levels were analyzed by quantitative real-time RT-PCR in triplicates. Values are given as mean ± SEM after normalization to RPL19. *P < 0.05 and **P < 0.01 compared with the corresponding shScramble clone (one-way ANOVA followed by Scheffè’s test). **b**, **c** The conditioned medium from RCC 786-O clones was tested for tube formation assay in vitro. In all, 15,000 Eahy.926 cells/well were incubated with 100 µl of conditioned media. Photographs were taken 18 h post incubation using an inverted microscope (magnification: 5 × ). Scale bar 400 µm. One representative picture for each clone of two independent experiments is shown. **d**, **e** Quantification of number of capillary/tube-like structures in **b** and **c**. Values are mean from two experiment ± SEM. Three independents wells were analyzed per experiment. *P*-value was determined by Kruskal–Wallis followed by a Dunn’s test. *P < 0.05 and ** = *P* < 0.01 compared with the corresponding shScramble clone. **f**, **g** RCC 786-O clones (10^6^ cells, each clone) were intradermal injected in the right flank of 6–8-weeks male NOD/SCID mice. Vehicle was injected in the left flank (contralateral flank). After 7 days, the skin was removed and photographs were taken under magnification glass. Pictures representative of each condition with similar results to the others of each group are shown. Scale bar 5 mm. **h**, **i** Quantification of vessel density calculated (number of vessels_cells side_ – number of vessels_vehicle side_)/Area. Results obtained from two independent experiments with four independent pictures for each condition are expressed as mean ± SEM. *P < 0.05 and **P < 0.01 compared with the corresponding Scramble clone (Kruskal–Wallis followed by a Dunn’s test)

Next, we studied RSUME contribution to early stages of VHL tumorigenesis. In particular, we focused on the angiogenic switch associated to an increase in VEGF, due to HIF transcriptional activity deregulation. In RCC 786-O clones expressing VHL, VHL_K171R_, VHL_L188V_ or VHL_L188V/K171R_, co-expression of shRNA against RSUME resulted in a decrease of VEGF mRNA, indicating that through the mechanism described above, RSUME impacts on VHL function (Fig. 5a). Similar results were obtained for VHL_Y112H/K171R_ and VHL_R167Q/K171R_ co-expressing RSUME shRNA clones (Supplementary Fig. S6).

To determine how these changes in VEGF may contribute to differential tumoral vessel formation, we first performed in vitro tube formation assays. When EA.hy926 endothelial cells were cultured in conditioned media of clones in which RSUME was silenced, there was a significant decrease in capillary-like structures formation (Fig. 5b–e and Supplementary Fig. S6). To study angiogenesis in a more complex context^26^, RSUME role on early angiogenic progress associated to VHL function was further demonstrated in an in vivo angiogenesis model. After 7 days, mice injected with stable clones expressing either VHL_WT_ or VHL_L188V_ presented new vessels around the injection area, but those clones in which RSUME expression was knocked-down showed a significant decrease in vessel density (Fig. 5f and h). This confirms, in vivo, that in absence of RSUME, VHL Type 2 mutant become more potent and might limit early tumoral angiogenesis. In accordance with the fact that RSUME impacts on VHL function independently of its sumoylation status, RSUME silencing resulted in a gain of function of both VHL_WT_ and VHL_L188V_ defective sumoylation variants (Fig. 5g and i).

## Discussion

**Fig. 6:**
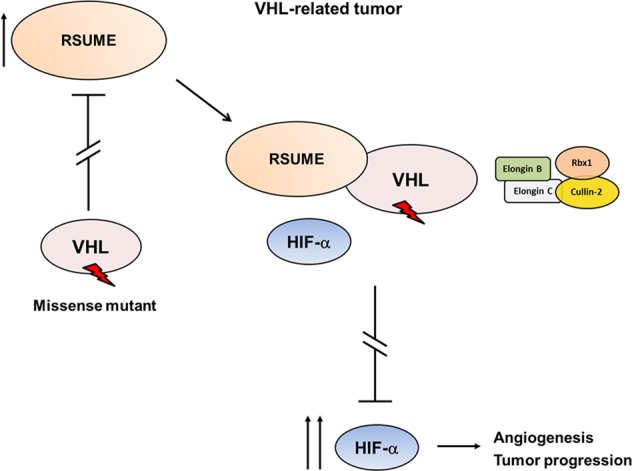
Proposed model for RSUME increase in VHL tumors and its action on VHL mutants function. VHL missense Type 2 mutants fail in negatively regulate RSUME levels, leading to a permissive context for increased RSUME in VHL-related tumors, like RCC. By protein–protein interaction with VHL mutants, RSUME reduces HIF-2α-VHL binding and negatively regulates the assembly of the ECV complex. By this mean, RSUME regulates VHL Type 2 mutants loss of function and worsens HIF transcriptional activity deregulation, thus leading to increased angiogenesis and tumor progression

RSUME was found to be expressed in several types of tumors with high angiogenic potential^17,27–30^. RSUME is expressed in tissues prone to tumor development in VHL syndrome^17^ and is upregulated in several tumors related to this disease^21^. High RSUME expression is associated with poor prognosis in RCC. Thus, RSUME might be a potential biomarker of the outcome of the disease. Our findings support a model in which RSUME is not downregulated in presence of VHL mutants, achieving high RSUME expression in specific tissues, were RSUME becomes critical for potentiating the missense VHL loss of function on HIF-2-α degradation, providing a permissive setting for the development of VHL tumors and promoting deregulated angiogenesis needed for tumor progression (Fig. 6).

Mutations in VHL generate reduction or absence of its function, which leads to a marked and unregulated amplification of HIF activity under non-hypoxic conditions^31^. There are mutant VHL isoforms that seem to behave similarly to wild-type VHL in terms of HIFα stabilization^12,13^ opening the question about the mechanisms by which these mutations impact on tumor phenotype^31^. Other alterations associated with VHL missense mutations, such as loss of PBRM1 or BAP1^11^, or changes in proteostasis of VHL^12^, have been proposed to support VHL pathological function. VHL has been identified as a multiadaptor protein, interacting with 117 different binding partners^32^. In this landscape RSUME emerges as a VHL modulator: it is expressed in VHL syndrome tumors, interacts with VHL mutants and promotes HIF-2α stabilization by disruption of the VHL interactions.

While VHL mutants are able to degrade HIF-α^12,13^, in presence of RSUME their ability to act on HIF-2 degradation worsens, displaying its loss of function. An in silico analysis of the effect of VHL mutations found in RCC samples shows that missense loss of function could be achieved by an impaired interaction between VHL mutants and interacting proteins^33^. In connection with this, several groups have demonstrated altered VHL-partners interactions^34,35^. The interaction of VHL mutants with HIF and RSUME show that RSUME overexpression disrupts HIF-2α-VHL mutants binding, shedding light onto the underlying mechanism behind HIF-2 stabilization. RSUME also decreases the recruitment of Elongin B and C to the complex. The reduced but still partial ability of some VHL mutants to bind to Elongin C and B has been shown to explain in part their capacity to still partially degrade HIF^13^. The protein–protein interaction of VHL mutants with RSUME impairs the ECV complex assembly contributing to the establishment of the mutant pathogenic phenotype.

Post translational modifications like ubiquitination and hydroxylation have been described as regulators of the oxygen-sensing system^31^. Particularly, dynamic sumoylation of HIF, involving SENP1^36^, has been implied in the regulation of HIF stabilization^17,36^. In addition to this, sumoylation of other components of the HIF system such as hypoxia associated factor (HAF) has been connected with the reprograming of HIF-2 response during VHL loss, needed for RCC tumor progression^37^. Sumoylation of VHL is relevant to affect its function as tumor suppressor^25,38^. Sumoylation/ubiquitination on Lysines 171 and 196 actively regulate VHL spatial distribution and decrease HIF-α inhibition^38^. We found that missense VHL proteins are sumoylated in presence of RSUME. In this context we tested if RSUME operates for its action on the loss of function of the mutants, through its sumoylation activity. Using a VHL sumoylation-deficient, which is mutated in Lysine 171, we observed that RSUME diminishes the binding between HIF and Type 2 VHL mutants defective in sumoylation and increases HIF-2α stability and transcriptional response, proving that this action of RSUME is mediated by a VHL sumoylation independent mechanism.

As already indicated 20.07% of 528 RCC samples showed high RSUME levels and a 23% decrease in survival rate. RSUME expression levels are higher in patients with VHL mutations, particularly in those in stage IV of the disease. Among those mutations, patients with missense VHL mutations showed increased RSUME levels. Both the lack of negative feedback compared to that exerted by normal VHL and the upregulation mediated by tumor hypoxia explain these higher levels of RSUME. The mutations create a permissive context for RSUME increased levels that regulate their loss of function and worsens the rate of survival of the patients. RSUME is not uniformly expressed in RCC tumors, in accordance with the intratumoral heterogeneity observed in RCC tumor samples^39–41^. Future pathological studies will be necessary to determine the relevance of its location.

One of the most relevant transcriptional targets of the HIF pathway in the tumoral context is VEGF.

Mutations in VHL that impact on its function generate a permissive setting for deregulation of VEGF^31^. As overproduction of VEGF promotes deregulated blood vessel formation^42^ and thus, high-vascularized tumors^43,44^, inhibitors that target VEGF and VEGF receptors (VEGFRs) have been used in the clinic^10^. Lately, therapies were also combined with novel HIF-2 activity inhibitors^6,9^. Resistance to these targeted therapies point to the need in understanding the molecular aspects that are involved in VHL disease setting. New approaches aim to impact on other crucial mediators^45,46^, which act on other signaling pathways that may provide escape routes that limit currently established treatments^47,48^. One strategy would be to act upstream HIF stability regulation, since HIF deregulation has been associated to the imbalance of factors that induce a proangiogenic scenario, thus triggering the tumoral angiogenic switch^49,50^. In line with this, a report in Hepatocellular Carcinoma tumor model showed decreased HIF-1 and 2α, VEGF and angiogenesis when wild-type VHL is upregulated^47^. RSUME binds missense VHL mutant proteins in normoxic conditions in which VHL is active and RSUME, mutant VHL and HIF-2α coexist in the same protein complex, suggesting that RSUME could act on HIF-2α deregulation by missense VHL mutants previous to the tumoral hypoxic state development. Silencing RSUME resulted in powerful wild-type and mutant VHL activity in early stages of angiogenesis switch, as shown in the in vitro tube formation and in in vivo angiogenesis assays. These results let us to suggest RSUME as a potential target to switch off angiogenesis in VHL-related tumors.

Taking together, our results reveal a protein–protein interaction mechanism involved in the VHL Type 2 mutants’ loss of function phenotype through the action of RSUME, both in vitro and in vivo. Supporting this, the bioinformatics analysis from TCGA dataset showing that RSUME increased levels are associated with VHL mutations and poor patients prognosis highlight the key role of RSUME as a possible biomarker of RCC VHL disease outcome. These findings hold the potential of adding a new target to those therapeutic strategies aimed to switch off angiogenesis in VHL tumors.

## Supplementary information

Supplementary Figure Legends

Supplementary Figure 1

Supplementary Figure 2

Supplementary Figure 3

Supplementary Figure 4

Supplementary Figure 5

Supplementary Figure 6

Supplementary Table 1

## References

[1] Maher ER, Neumann HP, Richard S (2011). von Hippel-Lindau disease: a clinical and scientific review. Eur. J. Hum. Genet..

[2] Nordstrom-O’Brien M (2010). Genetic analysis of von Hippel-Lindau disease. Hum. Mutat..

[3] Iliopoulos O, Kibel A, Gray S, Kaelin WG (1995). Tumour suppression by the human von Hippel-Lindau gene product. Nat. Med..

[4] Semenza GL (2013). HIF-1 mediates metabolic responses to intratumoral hypoxia and oncogenic mutations. J. Clin. Invest..

[5] Keith B, Johnson RS, Simon MC (2011). HIF1alpha and HIF2alpha: sibling rivalry in hypoxic tumour growth and progression. Nat. Rev. Cancer.

[6] Tarade D, Ohh M (2018). The HIF and other quandaries in VHL disease. Oncogene.

[7] Hu H (2014). Hypoxia-inducible factors enhance glutamate signaling in cancer cells. Oncotarget.

[8] Loboda A, Jozkowicz A, Dulak J (2010). HIF-1 and HIF-2 transcription factors–similar but not identical. Mol. Cells.

[9] Cho H (2016). On-target efficacy of a HIF-2alpha antagonist in preclinical kidney cancer models. Nature.

[10] Roskoski R (2017). Vascular endothelial growth factor (VEGF) and VEGF receptor inhibitors in the treatment of renal cell carcinomas. Pharmacol. Res..

[11] Miao D (2018). Genomic correlates of response to immune checkpoint therapies in clear cell renal cell carcinoma. Science.

[12] Yang C, Huntoon K, Ksendzovsky A, Zhuang Z, Lonser RR (2013). Proteostasis modulators prolong missense VHL protein activity and halt tumor progression. Cell Rep..

[13] Hoffman MA (2001). von Hippel-Lindau protein mutants linked to type 2C VHL disease preserve the ability to downregulate HIF. Hum. Mol. Genet..

[14] Gossage L (2014). An integrated computational approach can classify VHL missense mutations according to risk of clear cell renal carcinoma. Hum. Mol. Genet..

[15] Lai Y, Song M, Hakala K, Weintraub ST, Shiio Y (2011). Proteomic dissection of the von Hippel-Lindau (VHL) interactome. J. Proteome Res..

[16] Gao W, Li W, Xiao T, Liu XS, Kaelin WG (2017). Inactivation of the PBRM1 tumor suppressor gene amplifies the HIF-response in VHL-/- clear cell renal carcinoma. Proc. Natl Acad. Sci. USA.

[17] Carbia-Nagashima A (2007). RSUME, a small RWD-containing protein, enhances SUMO conjugation and stabilizes HIF-1alpha during hypoxia. Cell.

[18] Schulman BA (2011). Twists and turns in ubiquitin-like protein conjugation cascades. Protein Sci..

[19] Hay RT (2005). SUMO: a history of modification. Mol. Cell.

[20] Eisenberg-Lerner A, Ciechanover A, Merbl Y (2016). Post-translational modification profiling—A novel tool for mapping the protein modification landscape in cancer. Exp. Biol. Med. (Maywood)..

[21] Gerez J (2015). RSUME inhibits VHL and regulates its tumor suppressor function. Oncogene.

[22] Aranda E, Owen GI (2009). A semi-quantitative assay to screen for angiogenic compounds and compounds with angiogenic potential using the EA.hy926 endothelial cell line. Biol. Res..

[23] Cancer-Genome-Atlas-Research-Network. (2013). Comprehensive molecular characterization of clear cell renal cell carcinoma. Nature.

[24] Ricketts CJ (2018). The cancer genome atlas comprehensive molecular characterization of renal cell carcinoma. Cell Rep..

[25] Cai, Q. & Robertson, E. S. Ubiquitin/SUMO modification regulates VHL protein stability and nucleocytoplasmic localization. *PLoS ONE* **5**, e12636 (2010). 10.1371/journal.pone.0012636 PMC2936558 20844582

[26] Nowak-Sliwinska P (2018). Consensus guidelines for the use and interpretation of angiogenesis assays. Angiogenesis.

[27] Shan B (2012). RSUME is implicated in HIF-1-induced VEGF-A production in pituitary tumour cells. Endocr. Relat. Cancer.

[28] Ji CX (2018). MicroRNA-375 inhibits glioma cell proliferation and migration by downregulating RWDD3 in vitro. Oncol. Rep..

[29] He W (2017). Relationship between RSUME and HIF-1alpha/VEGF-A with invasion of pituitary adenoma. Gene.

[30] Chen X (2018). Knockdown of RWD domain containing 3 inhibits the malignant phenotypes of glioblastoma cells via inhibition of phosphoinositide 3-kinase/protein kinase B signaling. Exp. Ther. Med..

[31] Kaelin WG (2017). The VHL tumor suppressor gene: Insights into oxygen sensing and cancer. Trans. Am. Clin. Climatol. Assoc..

[32] Tabaro F (2016). VHLdb: A database of von Hippel-Lindau protein interactors and mutations. Sci. Rep..

[33] Razafinjatovo C (2016). Characterization of VHL missense mutations in sporadic clear cell renal cell carcinoma: hotspots, affected binding domains, functional impact on pVHL and therapeutic relevance. BMC Cancer.

[34] Lai Y, Song M, Hakala K, Weintraub ST, Shiio Y (2012). The interaction of the von Hippel-Lindau tumor suppressor and heterochromatin protein 1. Arch. Biochem. Biophys..

[35] Yang H (2007). pVHL acts as an adaptor to promote the inhibitory phosphorylation of the NF-kappaB agonist Card9 by CK2. Mol. Cell.

[36] Cheng J, Kang X, Zhang S, Yeh ET (2007). SUMO-specific protease 1 is essential for stabilization of HIF1alpha during hypoxia. Cell.

[37] Koh MY (2015). Hypoxia-induced SUMOylation of E3 ligase HAF determines specific activation of HIF2 in clear-cell renal cell carcinoma. Cancer Res..

[38] Cai Q, Verma SC, Kumar P, Ma M, Robertson ES (2010). Hypoxia inactivates the VHL tumor suppressor through PIASy-mediated SUMO modification. PLoS ONE.

[39] Jiang W (2016). Immunohistochemistry successfully uncovers intratumoral heterogeneity and widespread co-losses of chromatin regulators in clear cell renal cell carcinoma. PLoS ONE.

[40] Lopez JI (2016). Intratumor heterogeneity in clear cell renal cell carcinoma: a review for the practicing pathologist. APMIS.

[41] Zaldumbide L (2016). Snail heterogeneity in clear cell renal cell carcinoma. BMC Cancer.

[42] Kaelin WG (2009). Treatment of kidney cancer: insights provided by the VHL tumor-suppressor protein. Cancer.

[43] Jonasch E (2012). State of the science: an update on renal cell carcinoma. Mol. Cancer Res..

[44] Pierscianek D (2017). Study of angiogenic signaling pathways in hemangioblastoma. Neuropathology.

[45] Croci DO (2014). Glycosylation-dependent lectin-receptor interactions preserve angiogenesis in anti-VEGF refractory tumors. Cell.

[46] Saharinen P, Eklund L, Alitalo K (2017). Therapeutic targeting of the angiopoietin-TIE pathway. Nat. Rev. Drug. Discov..

[47] Iwamoto H (2015). Inhibition of hypoxia-inducible factor via upregulation of von Hippel-Lindau protein induces “angiogenic switch off” in a hepatoma mouse model. Mol. Ther. Oncolytics.

[48] Coleman ML, Ratcliffe PJ (2009). Angiogenesis: escape from hypoxia. Nat. Med..

[49] Hanahan D, Folkman J (1996). Patterns and emerging mechanisms of the angiogenic switch during tumorigenesis. Cell.

[50] Semenza GL (2000). HIF-1: using two hands to flip the angiogenic switch. Cancer Metastas-. Rev..

