# Supplementary material for: von Hippel-Lindau mutants in renal cell carcinoma are regulated by increased expression of RSUME
Source: Cell Death Dis. 2019 Mar 19;10(4):266. doi: 10.1038/s41419-019-1507-3 (PMC6424967; doi:10.1038/s41419-019-1507-3)
Supplement: Supplementary file 1 — Supplementary Figure Legends [file 41419_2019_1507_MOESM1_ESM.docx]

**SUPPLEMENTARY FIGURE LEGENDS**

**Supplementary Figure 1**

**RSUME blocks normoxia HIF-2α VHL-mediated degradation after hypoxia.**

COS-7 cells were transfected with 0.5µg of Flag-VHL, 0.5µg of V5-RSUME or its empty vector and 0.1µg of HA-HIF-2α expression vectors. 48h after transfection, cells were incubated for 4h in HPX and then harvested at indicated times of NMX. Cells extracts were analyzed by WB using the indicated antibodies. β-actin was used as a loading control. One representative experiment from three independent experiments with similar results is shown.

**Supplementary Figure 2**

**RSUME stabilizes HIF-2α by inhibiting VHL function independently of its sumoylation status.**

(A)COS-7 and (B) RCC-786-O cells were transfected with 0.5µg of Flag-VHL, 0.5µg of V5-RSUME or its empty vector and 0.1µg of HA-HIF-2α expression vectors. 48h after transfection, cells extracts were analyzed by WB using the indicated antibodies. One representative experiment from three independent experiments with similar results is shown. (C) COS-7 cells were transfected with 0.5µg of Flag-VHLK171R, 0.5µg of V5-RSUME or its empty vector and 0.1µg of HA-HIF-2α expression vectors. 48h after transfection, cells were incubated for 4h in HPX and then harvested at indicated times of NMX. Cells extracts were analyzed by WB using the indicated antibodies. β-actin was used as a loading control. One representative experiment from three independent experiments with similar results is shown. (D) COS-7 cells were transfected with 0.5 µg of V5-RSUME and/or 0.5 of µg Flag-pVHL or its empty vector, 0.3 µg of HA-HIF-2α and 0.1µg of Gam-1 or its control Gam-1MUT expression vectors. 48 h post-transfection, cells were lysed and the extracts were analyzed by WB using the indicated antibodies. β-actin was used as a loading control. One representative experiment from four independent experiments with similar results is shown.

**Supplementary Figure 3**

**K171R mutation on the VHL mutants impairs their sumoylation.**

COS-7 cells were transfected with 0.6 µg of each indicated Flag-pVHL variant, and/or 0.6 µg of 6xHis-SUMO-2, 0.1 µg of V5-Ubc9 expression vectors. 48h post-transfection cells were harvested, an aliquot of the lysates was directly analyzed by WB (Input) and the remaining extracts were used for Ni2+ affinity chromatography to purify 6xHis-SUMO-2 (Ni-NTA). Purified fractions were analyzed by WB using the indicated antibodies. One representative experiment from two independent experiments with similar results is shown.

**Supplementary Figure 4**

**RSUME acts on VHL disease variants with the K171R mutation.**

(A-C) COS-7 cells were transfected with 0.5 µg of Flag-VHL indicated variant and/or 0.5 µg of V5-RSUME expression vectors. 48h post-transfection cells were lysed and analyzed by WB using the indicated antibodies. β-actin was used as a loading control. One representative experiment from four independent experiments with similar results is shown.

**Supplementary Figure 5**

**RSUME with the Y61A/P62A mutation interacts with VHL.**

COS-7 cells were transfected with the following vectors: 0.5 µg of V5-RSUME and/or 0.5 µg V5-RSUMEY61A/P62A and 0.5 µg of indicated Flag-VHL variants. 48 h post-transfection cells extracts were Immunoprecipitated with anti-FLAG antibodies. Immunoprecipitated fractions and extract aliquots (Input) were analyzed by WB using the indicated antibodies. One representative experiment from three independent experiments with similar results is shown.

**Supplementary Figure 6**

**RSUME impacts on VHL mutants tubulogenesis.**

(a-b) RCC-786-O clones were lysed and VEGF mRNA levels were analyzed by quantitative real-time RT-PCR in triplicates. Values are given as mean ± SEM after normalization to RPL19. *P<0.05 and ***P<0.001 compared with the corresponding shScramble clone (Student’s *t*-test). (c-d) The conditioned medium from RCC-786-O clones were tested for tube formation assay in vitro. 15,000 Eahy.926 cells/well were incubated with 100 µl of conditioned media. Photographs were taken 18 h post-incubation using an inverted microscope (magnification: 5X). Scale bar 400µm. One representative picture for each clone of two independent experiments is shown. (e-f) Quantification of number of capillary/tube-like structures in panels c and d. Values are mean from two experiment ±SEM. Three independents wells were analyzed per experiment. *P<0.05 and **P<0.01 compared with the corresponding shScramble clone (Mann Whitney U test).

**Supplementary Table 1**

**Table used for Statistical analysis corresponding to Figure 5.**

RSUME FPKM values and stages for ccRCC tumor samples were accessed from The Human Protein Atlas. These data were merged with VHL mutational analysis from tumors with sequencing data available from Ricketts et al, 2018.
