# Supplementary figures and images for: von Hippel-Lindau mutants in renal cell carcinoma are regulated by increased expression of RSUME
Source: Cell Death Dis. 2019 Mar 19;10(4):266. doi: 10.1038/s41419-019-1507-3 (PMC6424967; doi:10.1038/s41419-019-1507-3)

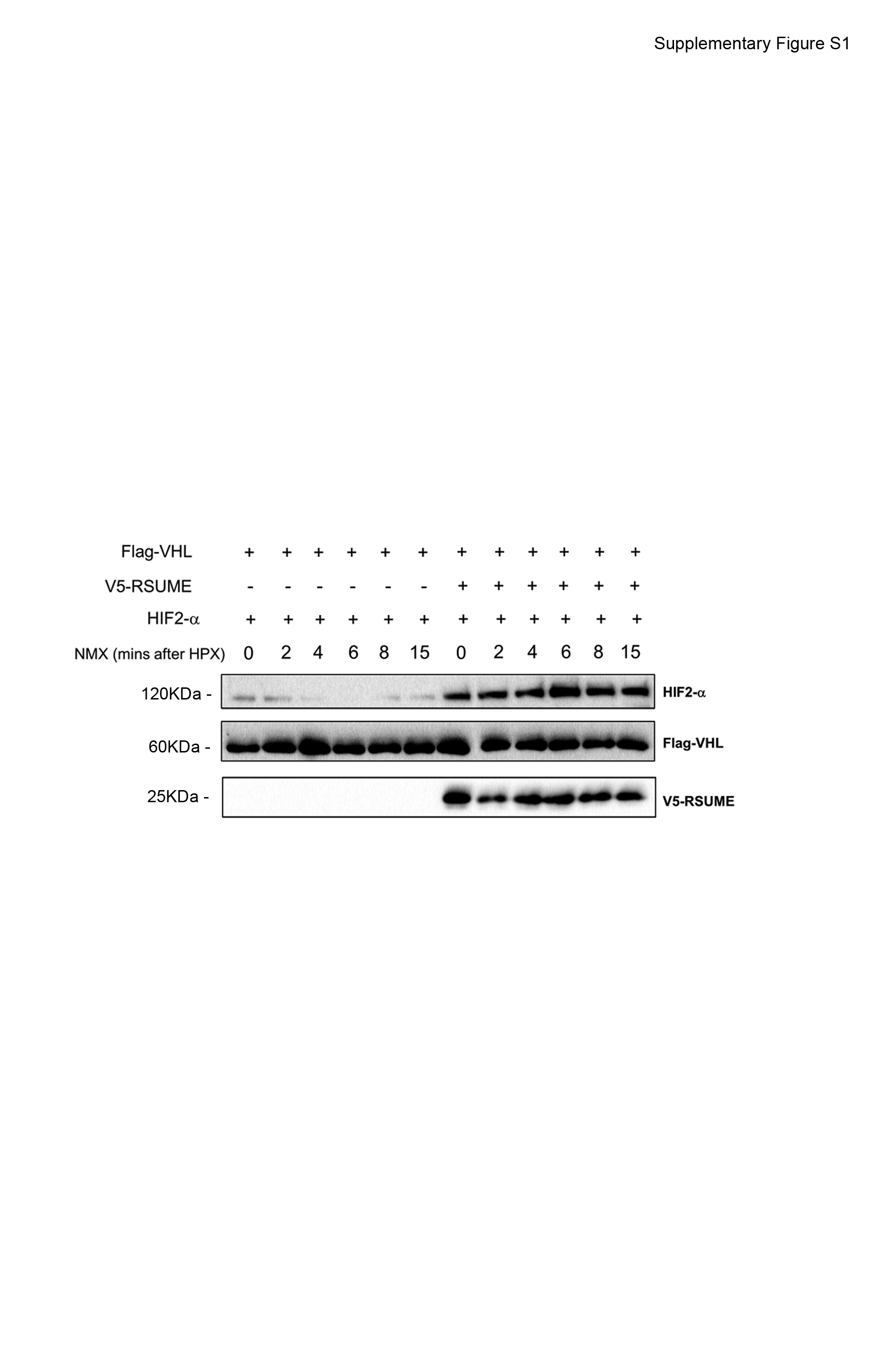

Supplement: Supplementary file 2 — Supplementary Figure 1 [file 41419_2019_1507_MOESM2_ESM.tif]

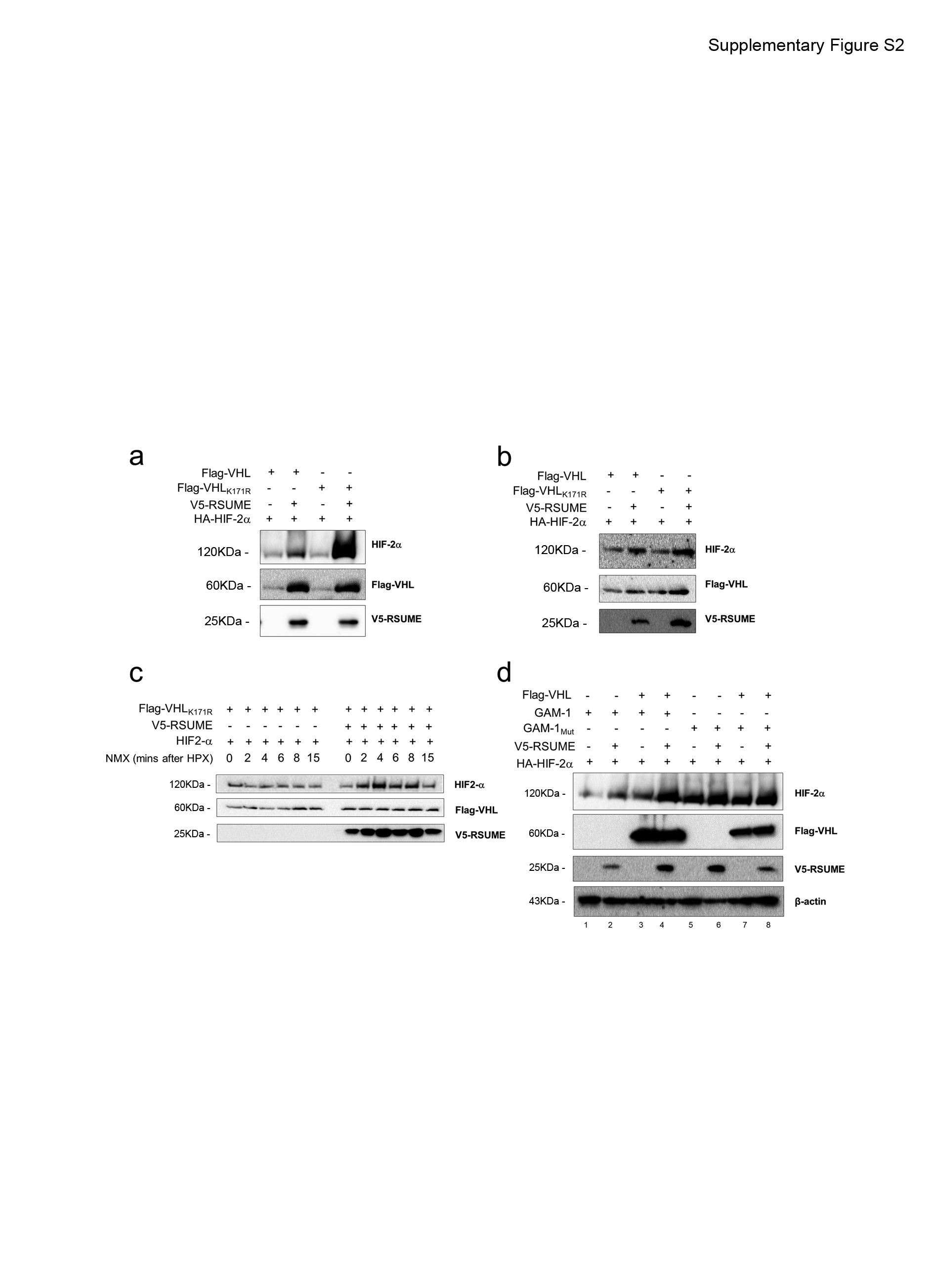

Supplement: Supplementary file 3 — Supplementary Figure 2 [file 41419_2019_1507_MOESM3_ESM.tif]

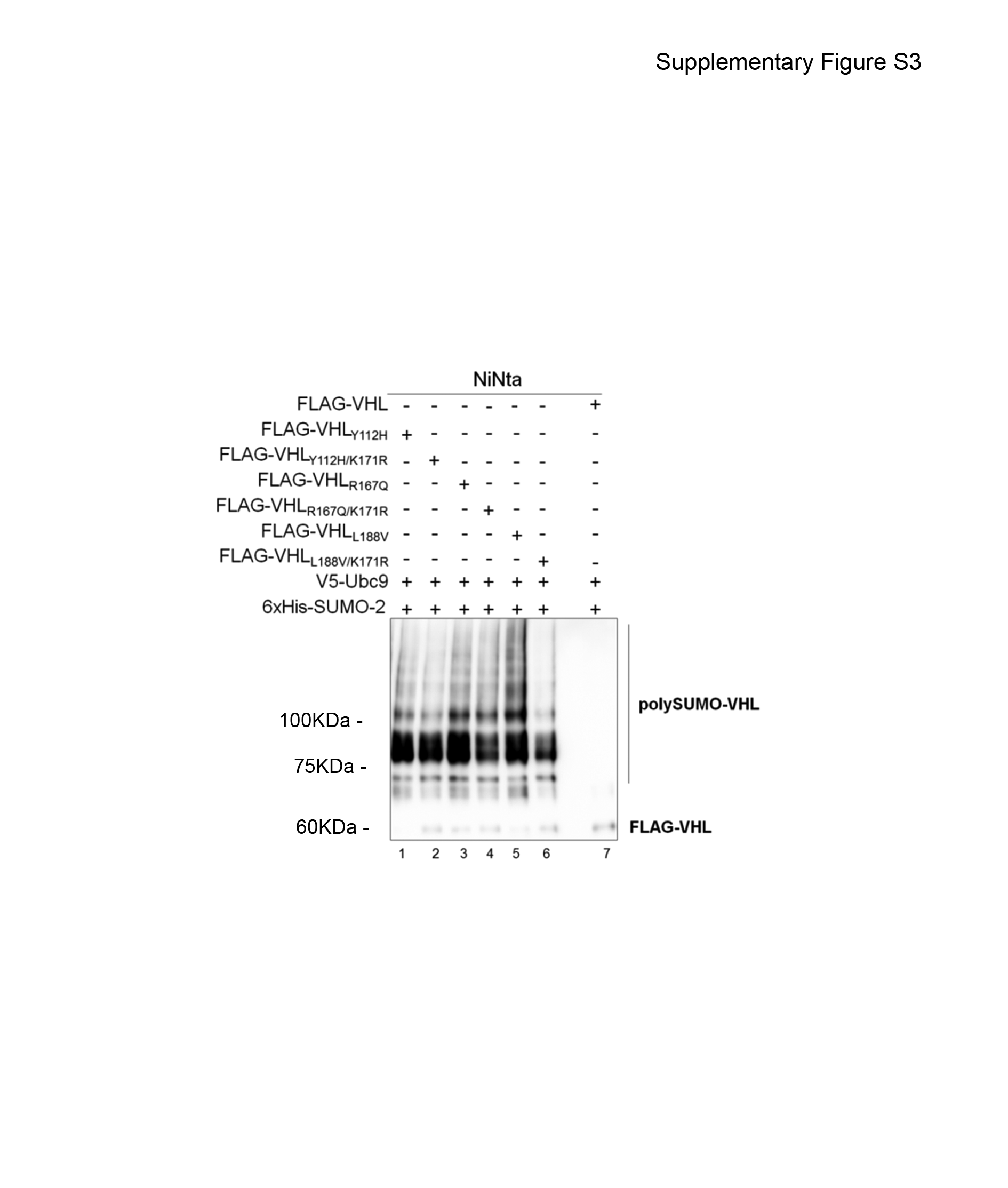

Supplement: Supplementary file 4 — Supplementary Figure 3 [file 41419_2019_1507_MOESM4_ESM.tif]

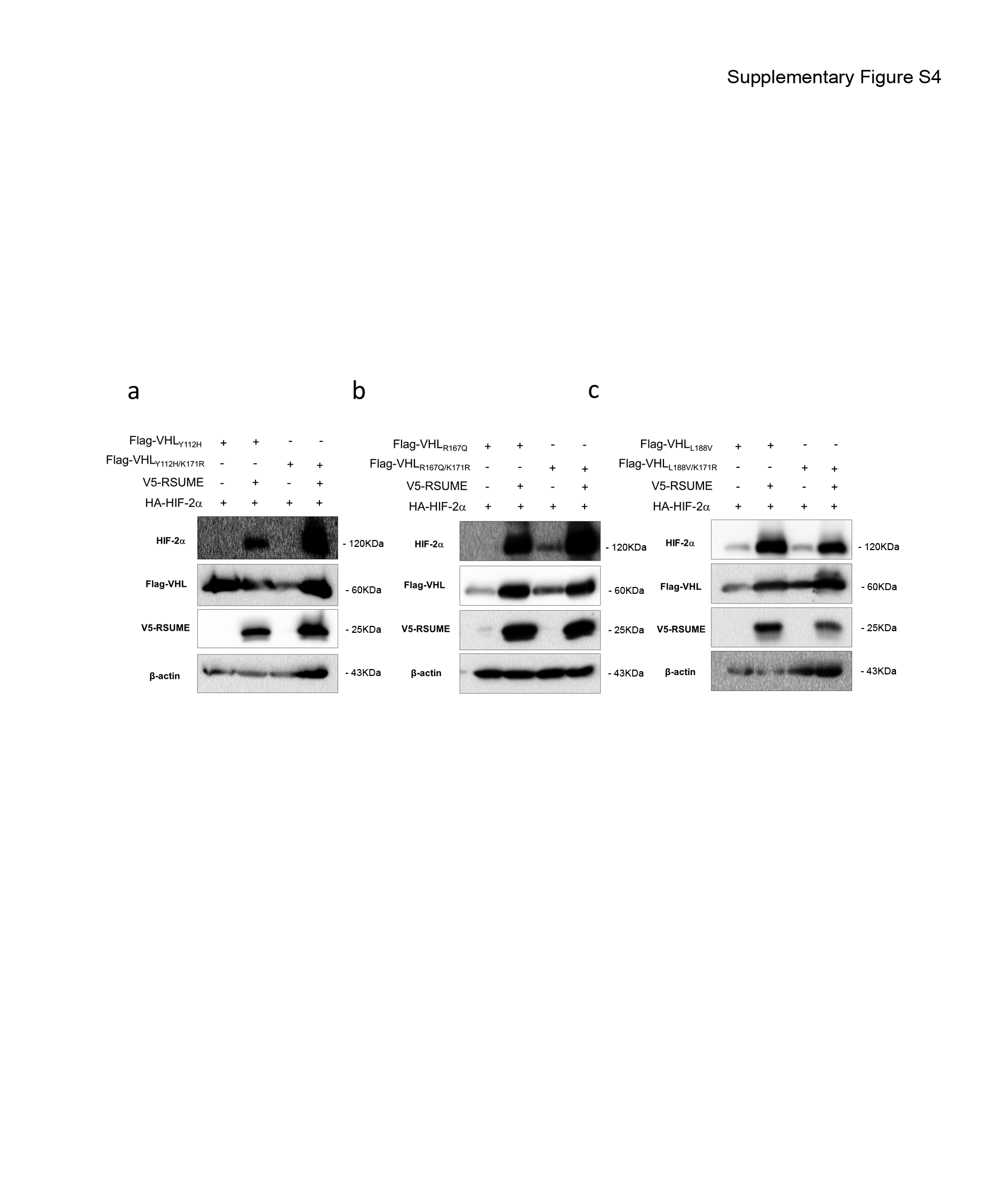

Supplement: Supplementary file 5 — Supplementary Figure 4 [file 41419_2019_1507_MOESM5_ESM.tif]

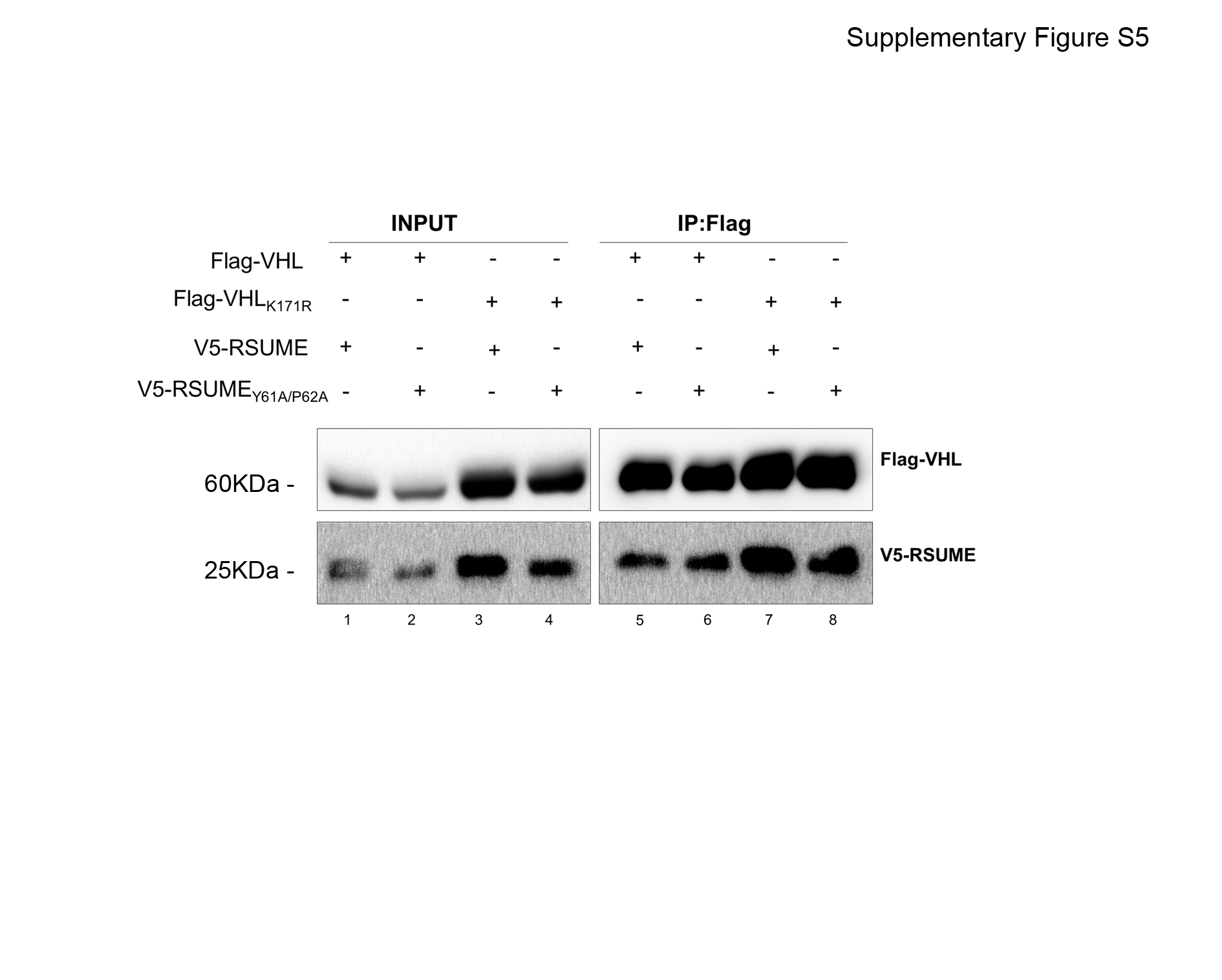

Supplement: Supplementary file 6 — Supplementary Figure 5 [file 41419_2019_1507_MOESM6_ESM.tif]

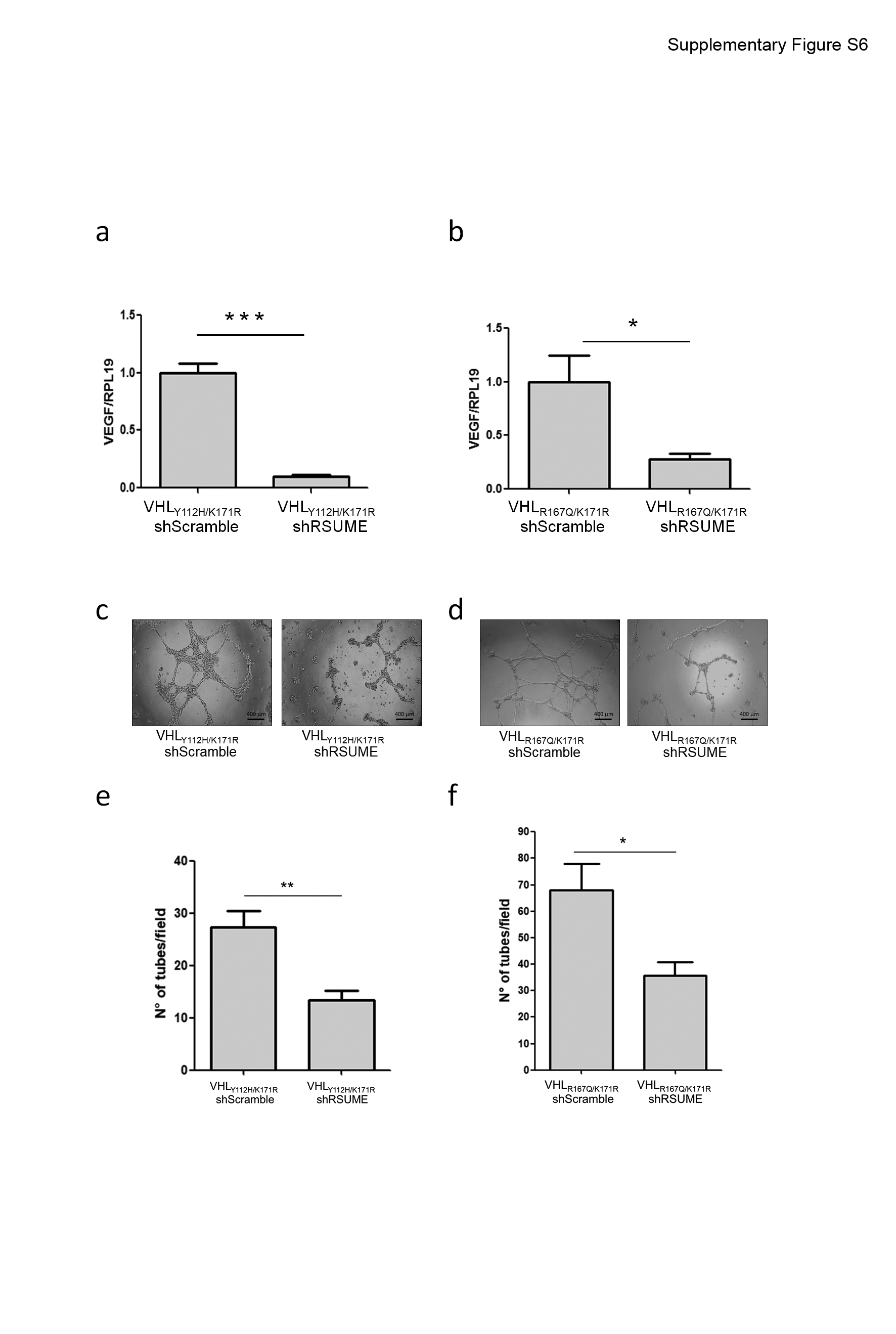

Supplement: Supplementary file 7 — Supplementary Figure 6 [file 41419_2019_1507_MOESM7_ESM.tif]
